# Does Student-Led Faculty Development Have A Place in Health Professions Education?

**DOI:** 10.15694/mep.2019.000034.1

**Published:** 2019-02-27

**Authors:** David Benjamin Wright, Ashley Mullen, Aimee Gardner

**Affiliations:** 1 Baylor College of Medicine

**Keywords:** faculty development, professional development, student-led teaching

## Abstract

This article was migrated. The article was marked as recommended.

**Background:** Instructors at health sciences universities typically transition directly from the clinical to academic setting, limiting their ability to formally develop proficiency in the science of teaching. We assess the effectiveness of a student-led faculty development course and faculty perceptions of the program.

**Methods:** Faculty from a clinical graduate program participated in a longitudinal student-led faculty development course. The course was offered in four separate 90-minute modules led by a student facilitator. Faculty completed course evaluations after each module, a knowledge assessment before and after the course, and a comprehensive evaluation upon completion of the course. Descriptive statistics were used to explore the effectiveness of the course and faculty perceptions.

**Results:** Faculty (N=5) exhibited increased knowledge in teaching and learning principles after the course (p < 0.01). The highest-rated area on the module evaluations was the relevance of the topics to the participants’ roles as instructors (4.31 ± 0.22). The lowest-rated area was pace of the modules (3.55 ± 0.62). The final course evaluation results showed that faculty rated the overall curriculum delivery very high (4.20 ± 0.46). Overall, faculty rated the student’s instructional quality at or above what they would expect from a peer faculty member (3.80 ± 0.72). Faculty expressed that the most valuable parts of the curriculum were applicable content, the introduction to evidence-based learning and teaching concepts, and the group discussions.

**Conclusions:** A student-led faculty development course improved faculty knowledge of learning, teaching, and assessment principles.

## Introduction

A key role of faculty at health sciences universities is to teach trainees, whether that instruction occurs in the classroom setting, clinical environment, or both. Even though instructors have developed competency in their clinical specialties, they are rarely given the opportunity to develop formal proficiency in the sciences of teaching, learning, or assessment. This lack of formal training has led many academic institutions to establish teaching and learning centers which provide pedagogical training for faculty through lectures, workshops, and observation opportunities (Center for Teaching Excellence). Studies have shown that the more resources institutions dedicate to faculty development, student learning outcomes and faculty satisfaction and engagement improve ( Gansemer-Topf and Schuh, 2003; Sherer, Shea and Kristensen 2003). In addition, a recent systematic review and meta-analysis confirms the effectiveness and positive impact of faculty development initiatives in healthcare institutions ( Bilal, Guraya and Chen, 2017). For these reasons, national accreditation bodies have recently revised their Common Program Requirements to include a requirement for faculty to be involved in faculty development initiatives at least annually (Accreditation Council of Graduate Medical Education).

Students are crucial stakeholders in the quality of teaching at health sciences universities, but are often excluded from faculty development efforts, despite their critical role in completing course and instructor assessments. Although literature regarding student participation in faculty development initiatives is limited, there have been reports describing efforts to incorporate student feedback in ongoing professional development activities ( Holdsworth, 2013) and to enlist students to teach instructors how to use technology ( Lang, Craig and Casey, 2017).However, the extent to which students can effectively train instructors in evidence-based teaching practices and develop ideas on how to implement them has not been examined. Given their frequency of encounters with instructors, and their role on the receiving end of instruction, students may be able to play a critical role in the faculty development process

To further explore these issues, we developed and implemented a student-led modular, longitudinal faculty development course for health professions education faculty. We sought to explore the feasibility and effectiveness of the course and to gather reactions from both the student and faculty regarding the credibility and impact of the student-led endeavor.

## Methods

### Development of curriculum

The course was organized into an introductory presentation and four subsequent 90-minute modules, taught over the course of five months. An overview of each module and associated goals and objectives are provided in Table 1. Delivery formats for each module can be found in Table 2.

**Table 1.** Content of the Four Course Modules

	Overview	Preparatory Assignment	Objectives
***Module 1: Culture***	The culture of a learning environment should be based on a correct understanding of how learning occurs in the human brain. This includes an acceptance of desirable difficulties and an understanding of the role of errors in the learning process.	Reading assignment regarding how learning occurs in the human brain	Understand the steps of the learning process Understand how desirable difficulties and a proper consideration of errors can enhance and improve the learning process
***Module 2: Learner***	A consideration of the individual learner’s experiences, interests, needs, and prior understanding of the material will result in better learning outcomes. Fostering a growth mindset in students will also contribute to better learning outcomes.	Video describing growth mindset	Differentiate between learner-centered teaching and teacher-centered teaching Evaluate how learner-centered teaching can improve the learning process Describe the effect of a growth mindset on a student’s learning
***Module 3: Methods***	Using a variety of evidence-based teaching methods in the classroom can improve the learning of the students.	Reading assignment regarding evidence-based retrieval methods	Differentiate between massed practice and other forms of retrieval practice Describe the evidence-based retrieval methods presented and use them in the classroom
***Module 4: Assessment***	A consideration of the role of different types of assessment and feedback in the learning process.	Reading assignment regarding the effect of frequency and timing of low-stakes quizzes in the classroom	Differentiate between formative and summative assessment Describe benefits of formative assessment Understand effect of frequency and timing tests Understand essential aspects of giving feedback to students

**Table 2.** Learning Strategies Used in Respective Modules

Module Topic	Learning Strategies Used				
	Spaced Retrieval	Generation	Reflection	Elaboration	Other Active Learning Strategies
***Module 1: Culture***					
How Learning Occurs in the Brain	x	x	x		x
Desirable Difficulties		x	x	x	
Addressing Errors		x	x	x	
***Module 2: Learner***					
Teacher-Centered Learning vs. Learner-Centered Learning		x	x		
Understanding Individual Learners				x	
Addressing Naïve Understanding	x	x	x	x	
Growth Mindset	x	x	x		x
***Module 3: Methods***					
Retrieval practice	x	x	x	x	x
Elaboration				x	x
Generation	x	x	x	x	
Reflection		x	x	x	
***Module 4: Assessment***					
Defining Assessment	x	x			
Formative assessment		x	x		x
Assessment Frequency and Placement	x	x	x		
Feedback		x		x	x

### Development of Assessment Tools

After the content of the course was developed, a knowledge test (Appendix 1) was completed by each faculty participant. The test consisted of twenty questions, five questions per module. This same test was taken by the faculty when the course was completed.

An evaluation was also completed by the faculty directly after each module. A five-point (1=very poor; 5=excellent) scale was used to rate indicators of module quality including the quality of the instructor, the relevance of the material, the pace of the module, and the effectiveness of the presentation. A five-point scale (1=not relevant at all; 5=crucially relevant) was also used to rate the relevance of each topic in the module to the faculty’s roles as instructors. Three open-response questions allowed the faculty to express their opinions related to what would change in their teaching because of what they had learned, what topics needed further clarification, and any other comments they had about the module.

Participants also submitted an 18-item course evaluation after the course. Ten items pertained to the effectiveness of the curriculum delivery based on best practices in teaching and peer coaching ( Huston and Weaver, 2007; Siddiqqi, Jonas-Dwyer and Carr, 2007; Skeff, 1992)using a 1 (“strongly disagree”) to 5 (“strongly agree”) Likert scale. Faculty also completed five items related to the student’s curriculum delivery and presentation skills as compared to performance expectations of faculty facilitators, using a Likert scale ranging from 1 (“significantly worse than what I’d expect from a peer faculty member”) to 5 (“significantly better than what I’d expect from a peer faculty member”). Finally, faculty were asked to provide their opinions via open response format about the most valuable part of the curriculum, ways in which they had applied concepts and strategies in their roles as an educator, how having a student facilitator for the material impacted their willingness or ability to apply the concepts, what advice they would have for others pursuing a student-led faculty development program, and any other comments about the curriculum.

### Analyses

Changes on the knowledge test were analyzed using basic descriptive statistics and a paired-samples t-tests. The results of the post-module course evaluations were compared to determine which modules were rated the highest and lowest, and which indicators of module quality were rated the highest and lowest. Thematic content analyses were used to identify potential themes in the open response items. All data were analyzed using SPSS version 24.0 (IBM; Chicago, IL) using an alpha of p<0.05. This project was deemed Quality Improvement, and thus IRB approval was not required.

## Results

### Participants

All faculty members (N=5) with core responsibilities in the orthotics and prosthetics (OP) program participated in this study. The average number of years since completing OP training was 6.40 (± 4.32), since being in an academic OP program was 4.40 (± 2.60), and since being faculty at this institution was 3.8 (± 1.91).

### Knowledge Test

The mean score on the pre-test was 9.20 ± 1.02 (n = 5). The highest baseline scores were seen in the learner and assessment areas, whereas the lowest scores were in the questions pertaining to culture and methods. The mean post-course score was 13.40 (± 0.92), an improvement of 4.20, or 21% (p < 0.01) from the pre-course test.

### Module Evaluations

Table 3 contains the ratings of each of the four modules, organized by indicators of module quality and relevance. The average overall rating for the modules was 4.10 (±0.15).

**Table 3.** Post-Module Evaluation Results (n=5)

Indicators of Module Quality	Instructor Ratings				
	Module 1	Module 2	Module 3	Module 4	Average
***Quality of Instructor***	4.00 (0.00)	3.60 (0.55)	4.40 (0.55)	4.20 (0.45)	4.05 (0.27)
***Relevance of Material***	4.00 (0.00)	3.80 (0.84)	4.40 (0.89)	4.00 (0.71)	4.07 (0.23)
***Pace of Module***	3.25 (0.50)	3.40 (1.52)	4.40 (0.55)	3.00 (1.23)	3.55 (0.62)
***Effectiveness of Module Presentation***	3.50 (0.58)	3.80 (0.45)	4.60 (0.55)	3.60 (0.55)	3.90 (0.29)
***Relevance of Topics to Role as an Instructor***	4.44 (0.52)	4.15 (0.42)	4.40 (0.23)	4.30 (0.20)	4.31 (0.22)
***Total Evaluation***	4.06 (0.16)	3.90 (0.38)	4.43 (0.55)	4.00 (0.20)	4.10 (0.15)

Values shown as means (standard deviations) 1 = very poor, 5 = excellent; 1 = not relevant at all; 5 = crucially relevant.

### Course Evaluation

Results of the final course evaluation are displayed in Table 4. Overall, faculty rated the curriculum delivery very high, with an overall mean of 4.20 (± 0.46).

**Table 4.** Final Course Evaluation

	Faculty Mean (SD)	% Agree or Strongly Disagree	% Disagree or Strongly Disagree
Established a positive learning climate in each session	4.20 (0.45)	100	0
Overall, ensured a safe, collegial environment where confidentiality was respected	4.80 (0.45)	100	0
Made clear that the aim of the curriculum was for development and improvement	4.60 (0.55)	100	0
Demonstrated control of the teaching sessions	3.80 (0.45)	80	0
Set goals for each session	4.60 (0.55)	100	0
Communicated goals for each session	4.00 (0.71)	80	0
Promoted understanding and retention of concepts	4.20 (0.84)	80	0
Provided feedback to participants	3.20 (0.84)	40	20
Promoted a climate of shared learning among facilitator and participants	4.60 (0.55)	100	0
Implemented tools to encourage self-directed learning	4.00 (0.71)	80	0
**Mean**	4.20 (0.46)	86	2
	**Faculty Mean (SD)**	**% Significantly Better Than Faculty or Better than Faculty**	**% Significantly Worse Than Faculty or Worse Than Faculty**
Demonstrated appropriate knowledge of concepts and material	3.60 (0.55)	60	0
Displayed confidence in instruction	2.40 (0.55)	0	60
Discussed topics of relevance to our roles	2.80 (0.84)	20	40
Demonstrated professionalism in instruction	3.00 (0.00)	0	0
“Practice what he preached” (i.e., implemented frameworks he was discussing/promoting)	3.20 (0.45)	20	0
**Mean**	3.00 (0.40)	20	20
**Total Mean**	3.80 (0.72)	64	8

Faculty ratings comparing the student instructor’s performance to the expected performance of a peer faculty instructor had an overall average of 3.00 (± 0.40). Faculty rated the student lower than what they would expect of a faculty instructor on only two items (“displayed confidence in instruction” and “discussed topics of relevance to your roles”), with all other items at or above what they would expect from a faculty instructor.

Themes from the open response items are displayed in Table 5. As shown, faculty overall had positive responses to curricular elements and methods of application. There was variability in faculty responses to having a student lead the course, ranging from it having no impact on their willingness to apply what they learned, to perceptions that the student has less expertise in this area because of their level of training and lack of empathy for faculty constraints.

**Table 5.** Final course evaluation open response items

Survey Item	Themes	Sample Comment
***Most Valuable Part of Curriculum***	Applicability	“Content and strategies presented applied to my own classroom scenarios and needs.”
	Introduction to evidence-based learning and teaching concepts	“A great introduction to teaching and learning theories and best practices.”
	Group discussions	“Sharing concrete ideas to overcome specific classroom barriers.”
***Methods of Application***	Low-stakes testing	“Scheduled assessments more frequently and count each one for less overall percentages.”
	Teaching approach and philosophy	“Being more cognizant of some of the methodologies and research has allowed me to consider different attitudes and approaches with faculty student interaction.”
***Impact of Having Student Facilitator on Ability or Willingness to Apply Concepts***	Limited expertise	“I believe it is inappropriate for a student who still requires faculty approval for projects, grades, and graduation requirements to stand up in front of the faculty to share ideas on how to improve instruction and assessment of our students.”
	No impact	“It didn’t impact my willingness to try to implement these concepts.”
	Student bias	“The presentations felt somewhat biased to the student’s own experience and learning framework.”
	Lack of appreciation for logistical constraints	“There was not an appreciation of some of the real limitations that exist in the program, faculty, and classroom.”
***Advice for Others Pursuing Student-Led Faculty Development Projects***	Tacit assumptions implied by having a student teach	“Learning specifically about educational techniques from a current student amounts merely to statements of dissatisfaction of existing educational approaches.”
	Involve faculty in planning and teaching	“Faculty and student should co-teach.”

## Discussion

Overall, the faculty in our study found the content very relevant, the module delivery very effective, and the environment safe and collegial. The scores on the knowledge test improved by an average of 21% after course completion. Some faculty had made small changes in their teaching by the end of the course, and most suggested that they would be making changes based on the course content in the future. Additionally, the faculty reported that the most valuable parts of the curriculum were applicable content (the relevance of the content was the highest rated indicator in the module evaluations), the introduction to evidence-based learning and teaching principles, and the group discussions. In sum, our findings suggest that a student-led faculty development course can achieve some of the same positive outcomes as other successful faculty development initiatives.

Despite the overall positive outcomes of the student-led curriculum, a few faculty voiced concerns about having a current student lead the initiative, pointing out potential awkwardness or credibility issues. Work in Australia aimed at creating opportunities for faculty and student dialogue to enhance teaching efficacy has documented similar phenomena ( Holdsworth, 2013). Specifically, some teachers found the process of having students engaged in conversations about teaching effectiveness confronting and even avoided participation in student-led sessions.” Thus, anyone seeking to pursue a student-led faculty development initiatives may be wise to proactively address these issues, or develop processes to combat barriers to buy-in among faculty.

Limitations of our study include a small sample size, including faculty from a single institution, and reliance on a newly developed curriculum and customized knowledge test. Future work should explore these phenomena with a larger sample to expand the generalizability of these findings. Additionally, the longitudinal nature of the curriculum delivery may have introduced confounders that could have impacted the relationships we explored. Directions for further research in this area may include students and faculty preparing and/or co-teaching a similar course, teaching such a course over a shorter period, and using other metrics to assess the effects of the course, such as pre/post instructor evaluations from students.

## Conclusions

This study demonstrated that student participation in faculty development can be beneficial to enhance baseline understanding of the science of teaching and learning, but measures may need to be taken to obtain political buy-in and ensure appropriate communication of intentions.

## Take Home Messages

• Student involvement in the faculty development process may be a win-win for both faculty and students.
• Faculty can enhance their understanding of teaching and learning concepts when taught by students.
• Those wishing to pursue student-led faculty development might need to consider faculty perceptions of student credibility.

## Notes On Contributors

D. Benjamin Wright, MS, is a recent graduate of the Orthotics Prosthetics Master’s program at Baylor College of Medicine in Houston, Texas.

Ashley Mullen, MSAT, CPO, LPO is an Assistant Professor and Associate Program Director in the Orthotics Prosthetics training program at Baylor College of Medicine in Houston, Texas.

Aimee K. Gardner, PhD, is Associate Professor and Assistant Dean at Baylor College of Medicine in Houston, Texas.

## Appendices

### Module 1 Questions

1. Research has indicated that there are three primary steps in the learning process. Please list the first step.

2. Which of the following describes the final step of the learning process?
  a. Reorganizing memory traces by connecting them to previous knowledge
  b. Creating cues that help recall the material when needed
  c. Converting sensory perceptions into meaningful mental representations
  d. Applying concepts to novel situations

3. Short term impediments in the learning process that make for stronger learning are called ________________________.

4. True or False: Research shows that errors in the learning process should be limited as much as possible in the early stages to prevent them from being learned.

5. Which of the following most closely describes the theory behind Error Management Training?
  a. Errors are a natural and inevitable result of active learning and can help learners determine how to improve, and therefore should be encouraged
  b. Errors do not contribute to the learning process, therefore the learning environment should be error-free
  c. Errors can be beneficial only after initial concepts are understood and encoded, and therefore early knowledge and skill acquisition phases should be error-free
  d. Errors are an inevitable result of active learning, but instructors should correct them as soon as possible before bad habits are acquired

### Module 2 Questions

1. A teacher-centered teaching approach is mostly associated with the transmission of knowledge from the ----------------_______________ to the _______________.

2. Which of the following is a correct description of SMART goals?
  a. Specific, measurable, accurate, referenced, time bound
  b. Specific, measurable, achievable, realistic, time bound
  c. Simple, measurable, achievable, realistic, time bound
  d. Simple, measurable, accurate, referenced, time bound
  e. Structured, measurable, achievable, realistic, time bound

3. True or False: Research suggests that learning improves when teachers customize their instructional strategies according to students’ learning styles.

4. Which of the following beliefs are aspects of a “growth mindset?” (circle all that apply)
  a. Effort is more important in overcoming challenges than mental capacity
  b. Effort is a sign of weakness
  c. Mistakes should be publicized in order to increase accountability
  d. A task performed poorly is a reflection of one’s inability
  e. Mistakes are opportunities to learn
  f. True or False: Praising students for their intellectual ability contributes to their “growth mindset”.

### Module 3 Questions

1. Cramming for an examination is an example of _______________ practice.

2. Practicing more than one type of problem or subject in a study session is referred to as ________________________.

3. “Elaboration” refers to which of the following learning methods? (circle all that apply)
  a. Writing a summary of new material in one’s one words
  b. Researching new material after class to learn more about the topic
  c. Relating new material to life outside of class
  d. Teaching the new material to someoneelse

4. Allowing students to attempt to solve a problem before they are taught how to solve it is an example of __________________.

5. All of the following are reasons that desirable difficulties should be incorporated in the learning process in and out of the classroom EXCEPT:
  a. They require students to exert more effort to learn
  b. They make learning more durable
  c. Students need to be in a calm, pleasant state for optimal learning
  d. They decrease a false sense of mastery of material

### Module 4 Questions

1. True or False: Summative assessment refers to cumulative evaluations used to measure student growth after instruction.

2. Which of the following are true of effective formative assessment? (circle all that apply)
  a. It provides instructors with feedback on how to make the learning process easier
  b. It reveals a student’s understanding of the material rather than ability to repeat facts
  c. It helps students see their progress
  d. It provides instructors with feedback regarding how to improve teaching and learning

3. Research suggests that tests should be administered _________ frequently to provide maximum benefit for learners.
  a. More
  b. Less

4. True or false: Feedback is most beneficial when provided in a positive - negative -positive format.

5. Good and well-timed feedback ______________ the positive effects and _______________ the negative effects of multiple-choice testing.
