# Supplementary material for: Does Student-Led Faculty Development Have A Place in Health Professions Education?
Source: MedEdPublish (2016). 2019 Feb 27;8:34. [Version 1] doi: 10.15694/mep.2019.000034.1 (PMC10712635; doi:10.15694/mep.2019.000034.1)
Supplement: Peer review history

# Reviewer report: Reviewer response for version 1

**DOI:** 10.21956/mep.19768.r29685

**Published:** 2019-03-07

**Authors:** Tan Nguyen

**Affiliations:** 1 Deakin University

This review has been migrated. The reviewer awarded 2 stars out of 5 This paper presented a novel approach to faculty development through a student-led intervention. Despite the evaluated improvement in knowledge gain by participants, caution should be emphasised on the appropriateness of student-led intervention, which requires medical education expertise in the development of the course content, and potential lack of teaching skills by students to optimise learning. (This includes both formal learning at a postgraduate program and learning from experience). The outcomes only reflected on Level 1 outcomes based on Kirkpatrick's Model, therefore, the intervention impact may be limited long-term. And as also previously mentioned, the low numbers of participants mean the results cannot be generalisable. The credibility of the student-led intervention should be explored further.

Reviewer Expertise:

NA

No decision status is available

# Reviewer report: Reviewer response for version 1

**DOI:** 10.21956/mep.19768.r29687

**Published:** 2019-03-03

**Authors:** Megan Anakin

**Affiliations:** 1 University of Otago

This review has been migrated. The reviewer awarded 3 stars out of 5 Great to see an initiative where students instruct their teachers. This article addresses the bidirectional nature of teaching and learning, issues of power, psychological safety, and cultural expectations. By culture, I mean the culture of medical education and how the practice of medicine has established roles about what is traditionally expected of teachers and students. In this well-written article, the authors have established and explained the need for this research. The intervention is described sufficiently to see the linkages among the measuring instruments and methods used to evaluate and study it. The low sample size limits the statements that the authors can make about the generalisability of the results, however, there are other gems in the results. As expected, faculty learned as shown by the pre- and post-testing. While these results are not surprising, the interesting findings are in relation to “student credibility” as teachers. This gap in ‘expectations of who is a teacher’ held by faculty and in ‘confidence to teach’ reported by students merits further investigation. I look forward to seeing the next steps these authors pursue regarding this interesting topic.I have two suggestions that the authors may wish to consider to improve the article. In the abstract, a p-value stated would be enhanced by explaining other features of the difference pre- and post-test. (Please see read the following article about reporting p-values: Wasserstein RL, Lazar NA. The ASA’s statement on p-values: context, process, and purpose. The American Statistician. 2016 Mar 7;70(2):129-33.). There are missing data in Table 4 because the percentages shown do not total 100. Please consider stating the category/descriptor for the missing data and present those data too. The complete set of results will help the reader make better sense of the results that are reported. For helpful suggestions about presenting results in conventional tabular format, please see guidelines such as https://www.apastyle.org/.

Reviewer Expertise:

NA

No decision status is available

# Reviewer report: Reviewer response for version 1

**DOI:** 10.21956/mep.19768.r29689

**Published:** 2019-03-01

**Authors:** Subha Ramani

**Affiliations:** 1 Harvard Medical School, Brigham and Women's Hospital

This review has been migrated. The reviewer awarded 4 stars out of 5 I enjoyed reading this paper that presents a slightly new twist on faculty development. I agree that as the principal stakeholders, students should have a role in teaching faculty how to teach. This is a well designed small study and well written manuscript. Throughout the paper, I had no trouble following the aims, methods, evaluation and implications.Neatly done.I agree with the limitations that the sample was very small, this could be a pilot which leads to a larger study.This can be done at multiple institutions which have faculty development programs to strengthen the implications.I would also recommend that the next phase should involve some revisions to the curriculum:- students and faculty co-teach so each can view the other's perspective and learn from each other- The modular content is fine. Under culture, I would like to see the institutional culture, psychological safety and institutional mindset addressed. Under methods, I hope that participants are not only learning about how to gather evidence, but also application of evidence to a variety of teaching methods (large group, small group, case based, clinical, simulation, technology etc). I did not see workplace assessment and hope the assessment module includes that.- Evaluation of the curriculum currently includes Level 1 and 2 of Kirkpatrick pyramid with some self-reports of intended behavior change. In the next iteration, this can be more formally assessed by students / peer coaches etc.Well done and I hope the authors continue this work and teach the rest of the health professions education world.

Reviewer Expertise:

NA

No decision status is available

# Reviewer report: Reviewer response for version 1

**DOI:** 10.21956/mep.19768.r29688

**Published:** 2019-03-01

**Authors:** Bonnie Taylor

**Affiliations:** 1 University of Texas Health Science Center - San Antonio

This review has been migrated. The reviewer awarded 3 stars out of 5 The problem as stated explicitly refers to the need for clinical instructors to gain knowledge in effective pedagogy, but also implies a need for gaining experience. The faculty development course as described appears to give benefit to the participants for increasing knowledge concurrent with similar benefit to the student instructors in skill-building. This interpretation of the problem appears to support the stated research purpose: "We sought to explore the feasibility and effectiveness of the course." In education, as in medicine, expertise matters: effectiveness of teaching is somehow related to expertise in the clinical (classroom) environment, is it not? If the selected intervention was indeed novel, perhaps the authors would consider including further description of how the student-teacher-experts (or proficients) were selected and prepared for the experience. This might provide the reading audience some indication of the feasibility for implementing such a course design, and would give readers further information for interpreting study findings.

Reviewer Expertise:

NA

No decision status is available

# Reviewer report: Reviewer response for version 1

**DOI:** 10.21956/mep.19768.r29686

**Published:** 2019-02-27

**Authors:** Felix Silwimba

**Affiliations:** 1 University of Lusaka

This review has been migrated. The reviewer awarded 4 stars out of 5 this a good informative health professional study. it applies across all regions of the world. the role of students is often times neglected.

Reviewer Expertise:

NA

No decision status is available
